# Case Report: The role of nitric oxide inhalation and prone positioning ventilation in oxygen improvement in patients with refractory hypoxemia following acute type A aortic dissection surgery: two cases report and literature review

**DOI:** 10.3389/fcvm.2025.1627953

**Published:** 2025-07-04

**Authors:** Xiaofang Wang, Kangwei Sun, Weijun Yang, Xia Shao, MinJian Kong, Wei Cui, Lixue Wang, Gensheng Zhang

**Affiliations:** ^1^ Department of Cardiovascular Surgery, Second Affiliated Hospital, Zhejiang University School of Medicine, Hangzhou, China; ^2^ Department of Critical Care Medicine, Second Affiliated Hospital, Zhejiang University School of Medicine, Hangzhou, Zhejiang, China; ^3^ Department of Emergency Medicine, Dongyang People’ Hospital of Wenzhou Medical University, Dongyang, Zhejiang, China; ^4^ Department of Breast Surgery, The First Affiliated Hospital, Zhejiang University School of Medicine, Hangzhou, China; ^5^ Key Laboratory of Multiple Organ Failure (Zhejiang University), Ministry of Education, Hangzhou, Zhejiang, China; ^6^ Department of Critical Care Medicine, Taizhou Hospital of Zhejiang Province Affiliated to Wenzhou Medical University, Taizhou, China

**Keywords:** nitric oxide, prone positioning, aortic dissection, hypoxemia, case report

## Abstract

Refractory hypoxemia following Acute Type A Aortic Dissection (ATAAD) surgery presents a significant clinical challenge. How to improve the refractory hypoxemia after ATAAD surgery is very critical for achiving perfect outcome of these patients. We report two cases of severe hypoxemia after ATAAD surgery that improved with inhaled nitric oxide (iNO) and prone positioning ventilation (PPV) therapy, despite traditional treatments including ventilator parameter optimization, performance of recruitment maneuvers, administration of glucocorticoids, diuresis and/or CRRT used for maintenance of a negative fluid balance, and secretion aspiration via fiberoptic bronchoscopy. Finally, these two patients were successful weaned from mechanical ventilation and subsequent discharge from the hospital.We provide the successful experience in the application of combination treatment of iNO and PPV in patients with refractory hypoxemia after ATAAD surgery, which will pave the way for the following randomized controlled clinical trials.

## Introduction

ATAAD is a lethal cardiovascular disease involving the ascending aorta, characterized by abrupt onset, critical progression, and a high mortality rate (1). Emergency surgery represents the preferred treatment for ATAAD (2). However, patients exhibit a notably high incidence of postoperative complications. Hypoxemia is one of the severe complications following ATAAD surgery, with an incidence rate as high as 30%–50% (3, 4). When traditional therapeutic approaches prove insufficient, PPV has emerged as an effective modality for hypoxemia management. The literature reports that PPV not only improves oxygenation in Acute Respiratory Distress Syndrome (ARDS) but also reduces mortality (5). However, in ARDS patients following aortic dissection surgery, PPV may carry risks such as impaired wound healing, hemodynamic instability, bleeding, catheter dislodgement, and even cardiac arrest (6). Therefore, the application of PPV in post-operative ARDS patients with type A aortic dissection should be approached with caution. In recent years, multiple studies (7–9) have reported the use of iNO in ARDS after aortic dissection surgery, demonstrating its effectiveness in improving oxygenation without significant side effects, though it does not reduce mortality. We hypothesize that combining PPV with iNO may have a synergistic effect, enhancing therapeutic efficacy while reducing adverse complications. Currently, there are no reported cases of combined PPV and iNO therapy in ARDS patients following type A aortic dissection surgery. Herein, we report two cases of severe hypoxemia after ATAAD surgery treated with iNO and PPV.

## Case presentations

### Case 1

A 46-year-old male patient weighing 130 kg with BMI 40 was admitted with “back pain for 1 day.” CT aortography demonstrated a Stanford Type A dissecting aortic aneurysm involving the thoracoabdominal aorta. Emergency surgery was performed, including median ascending aortic replacement, aortic valvuloplasty, and aorta-carotid artery bypass grafting. The patient had comorbidities of hypertension and diabetes mellitus. Preoperative findings included massive pericardial effusion, cardiac tamponade, elevated serum creatinine, and acute kidney injury (AKI).

**Figure 1:**
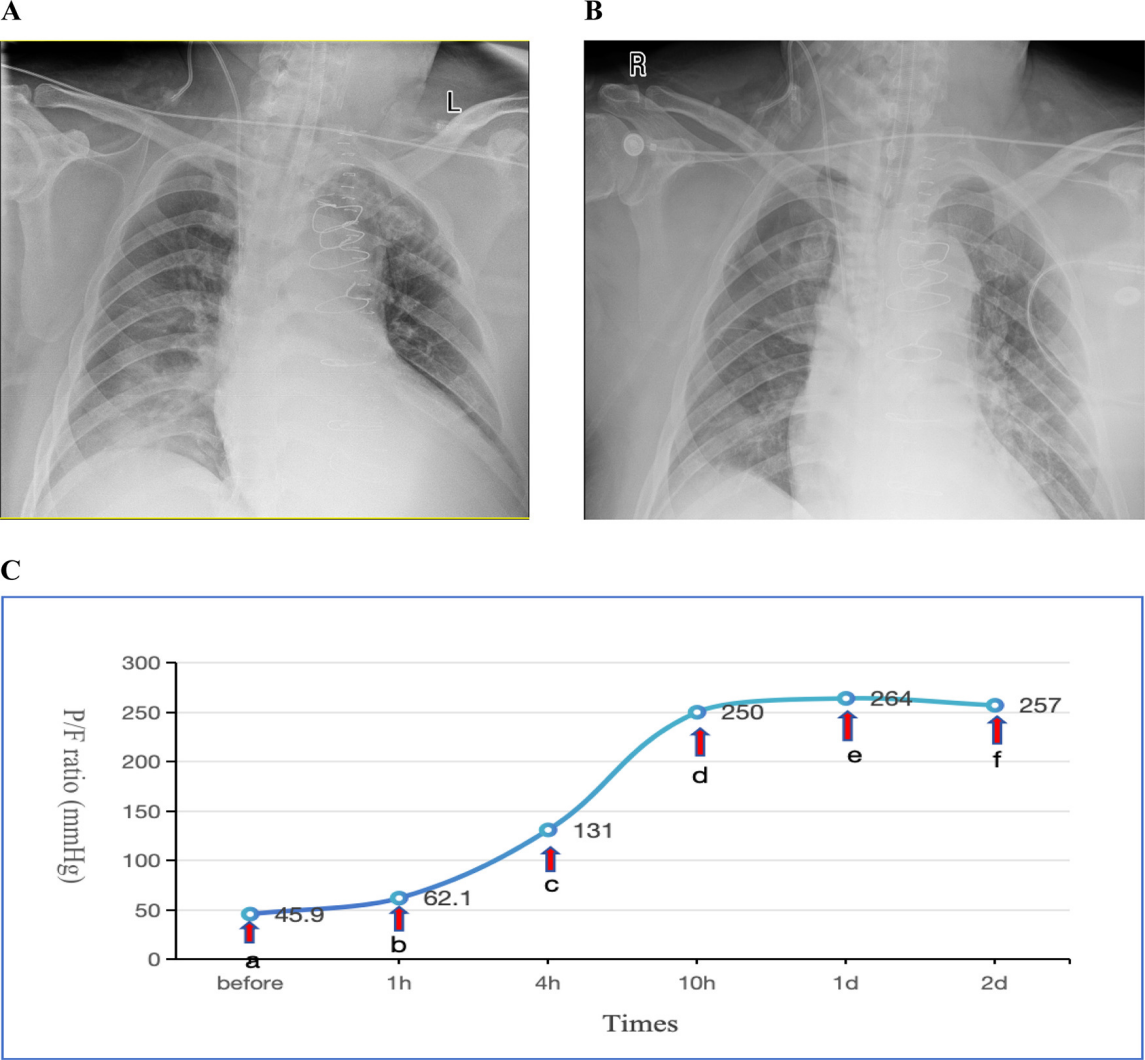
**(A)** Chest x-ray obtained one day prior to iNO showing a bilateral pulmonary infiltration; **(B)** chest x-ray performed after 3 days of iNO therapy showing no significant changes; **(C)** changes in oxygenation index (P/F) before and after interventions at indicated timepoints. Before (a) iNO; (a,b) iNO + PPV; (b,c) iNO + PPV + FB; (c–f) iNO. iNO, Inhaled nitric oxide; PPV, prone posioning ventilaton; FB, Fiberoptic bronchoscopy.

Following surgery, the patient was transferred to the intensive care unit (ICU) with stable hemodynamic parameters and regained consciousness after the cessation of sedation. The patient developed severe AKI characterized by oliguria and hyperkalemia, necessitating the initiation of continuous renal replacement therapy (CRRT) on the day of ICU admission. Upon admission to the ICU, the patient's oxygenation index (PaO_2_/FiO_2_) was 342 mmHg, but it gradually declined over time. On the first postoperative day, the oxygenation index (OI) fluctuated between 96 and 126 mmHg. There was no clear evidence of infection, and the decline was attributed to factors such as the patient's high body weight, aortic dissection surgery under cardiopulmonary bypass, minor pulmonary effusion, atelectasis, and ventilation-perfusion mismatch. Due to excessive pericardial and mediastinal drainage output and the patient's status post median sternotomy on the first postoperative day, ongoing CRRT treatment, and a high body weight (BMI 40), prone positioning ventilation was temporarily deferred. Instead, ventilator parameters were adjusted, with positive end-expiratory pressure

(PEEP) set between 10 and 15 cmH_2_O. Methylprednisolone 40 mg IV push twice daily (bid). For infection prophylaxis, clindamycin 0.6 g bid was administered during the first two postoperative days. By the second postoperative day, the OI still showed no improvement, and there remained no obvious signs of infection. Sputum production was not significantly increased, and inflammatory markers showed no notable upward trend. Drainage output had significantly decreased compared to the first postoperative day. INO therapy was initiated at a dose of 10 ppm based on Methylprednisolone 40 mg IV push twice daily (bid), leading to a marked improvement in oxygenation as evidenced by an increase in SpO_2_ from 88% to 98% within 30 min of administration. However, on the third postoperative day, the patient's OI deteriorated sharply, reaching a critical low of 45.9 mmHg. Vital signs showed a body temperature of 37.2°C. Endotracheal suction yielded minimal secretions. Physical examination revealed coarse breath sounds bilaterally, with diminished sounds on the right side. Bedside chest x-ray demonstrated bilateral minimal pleural effusions, right lung infiltrates, and partial resolution of right-sided effusion (Figure 1A). Echocardiography showed an LVEF of 60%, ruling out cardiogenic pulmonary edema. Inflammatory markers showed: WBC: 9.1 × 10^9^/L, Neutrophils:85.1%, IL-6:17.3 pg/ml, CRP:174.3 mg/L (all showing downward trends from previous measurements). Given these findings, the patient was diagnosed with severe ARDS according the Berlin criteria. Immediate prone positioning ventilation was initiated while maintaining iNO therapy at 10 ppm. Intravenous (IV) push of 80 mg Methylprednisolone Sodium Succinate was implemented at the same time. Piperacillin-tazobactam 4.5 g intravenously every 8 h (q8h) was initiated for pulmonary infection prophylaxis. Despite an acute renal failure diagnosis (creatinine clearance: 31.28 ml/min), the standard dose was maintained based on the patient's weight. After 1 h of prone posioning ventilation, the oxygenation index improved to 62.1 mmHg. However, by 2 h, significant ventilator dyssynchrony developed with elevated peak airway pressures (reaching 35 cmH_2_O), given suspected airway obstruction by secretions, necessitating return to supine position. Subsequent fiberoptic bronchoscopy revealed copious retained secretions at the orifices of the bilateral main bronchi, which were thoroughly suctioned. Following PPV, bronchial toilet and continuation of iNO therapy, although the Chest x-ray showed no significant differences between one day prior to nitric oxide inhalation (iNO) and after 3 days of iNO therapy (Figures 1A,B), the patient's oxygenation showed marked clinical improvement, which was shown in detail in Figure 1C. Intermittent prone positioning and nitric oxide inhalation therapy were maintained until tracheal extubation on postoperative day 16, when the patient's OI was 278 mmHg. Renal function recovery was protracted, necessitating intermittent CRRT over 31 days. Following family requests for continued ICU care, the patient was discharged for rehabilitation after 37 days of hospitalization.

### Case 2

A 51-year-old male patient (weight: 75 kg, BMI: 26) was admitted with a chief complaint of “chest and back pain persisting for over 3 h.” CT angiography of the thoracic and abdominal aorta confirmed a dissecting aortic aneurysm involving the thoracoabdominal aorta (DeBakey Type I, Stanford Type A). Emergency surgical interventions were performed, including hemiarch replacement, aortic valvuloplasty, and partial ascending aortic resection with synthetic graft replacement. The patient had a history of hypertension.

**Figure 2:**
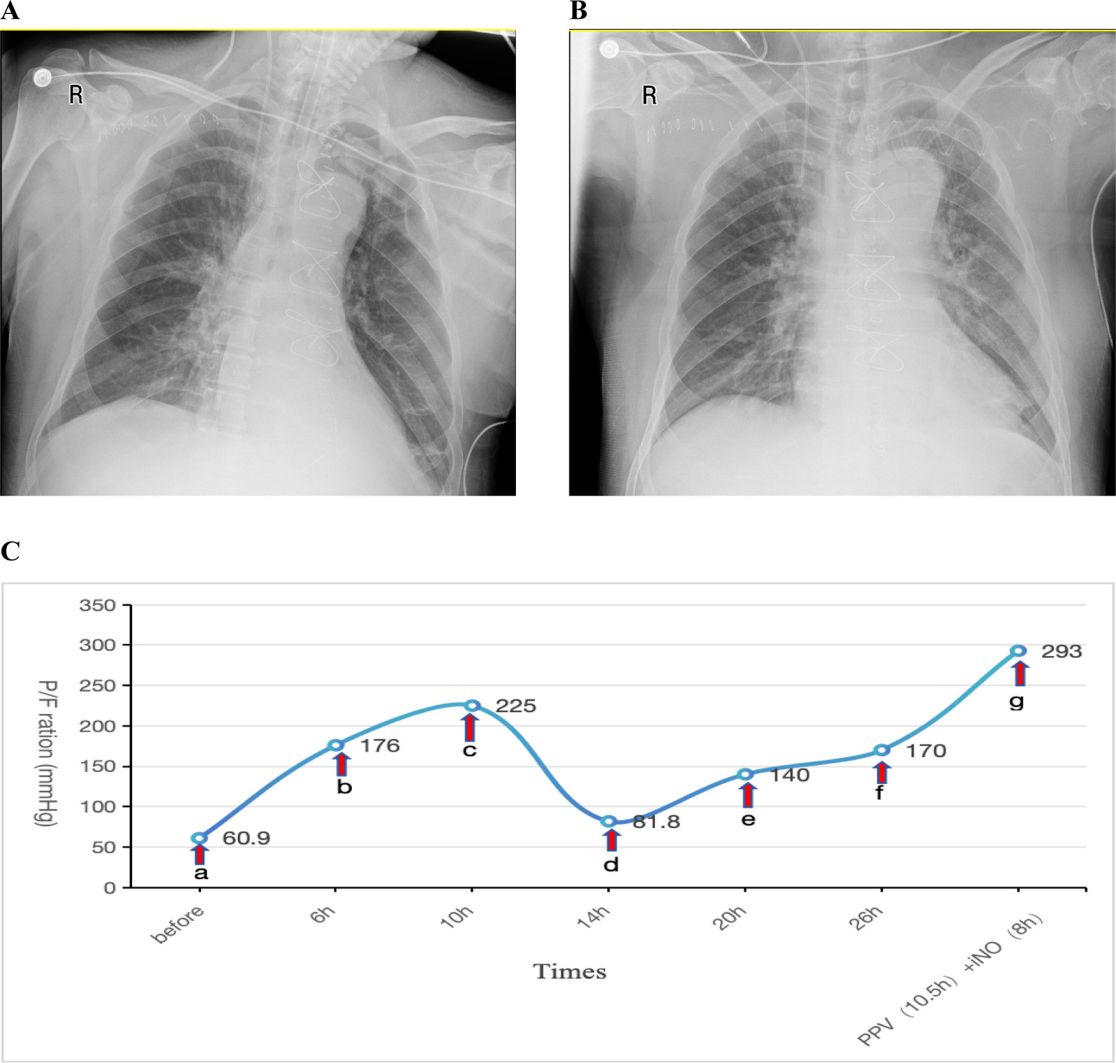
**(A)** Chest x-ray obtained one day prior to iNO showing a bilateral pulmonary infiltration; **(B)** chest x-ray performed after 2 days of iNO therapy showing no significant changes; **(C)** changes in oxygenation index (P/F) before and after interventions at indicated timepoints. (a–c) PPV; (c,d) SPV; (d–f) iNO; (f,g) PPV (10.5 h), iNO (8 h). iNO, inhalation of nitric oxide; PPV, prone posioning ventilaton; SPV, supine posioning ventilaton.

Postoperatively, the patient was transferred to the ICU with an initial P/F ratio of 183 mmHg and mild renal dysfunction (elevated serum creatinine). During the first two postoperative days, the patient maintained acceptable oxygenation. Therapeutic interventions included: Clindamycin 0.6 g bid for infection prophylaxis, Methylprednisolone 40 mg IV push once daily (qd) for anti-inflammatory management, Furosemide to maintain negative fluid balance.Ventilator settings remained elevated, with FiO_2_ fluctuating between 50%–80% and PEEP at 10–12 cmH_2_O. A spontaneous breathing trial (SBT) was attempted for weaning and extubation but failed. On postoperative day 3, oxygenation significantly deteriorated (P/F: 60.9 mmHg). A Chest x-ray showed a bilateral pulmonary infiltration (Figure 2A). Echocardiography indicated an LVEF of 57%, excluding cardiogenic pulmonary edema. This patient was also diagnosed with severe ARDS according the Berlin criteria.Despite conventional therapies mentioned above and the prophylactic antibiotic regimen was escalated to intravenous piperacillin-tazobactam (4.5 g q8h) for prevention of ventilator-associated pneumonia, refractory hypoxemia persisted. The patient remained hemodynamically stable with a heart rate of 70 bpm and blood pressure of 121/63 mmHg, maintaining circulation without vasoactive agents. The drainage output averages 20 ml/h. The patient also had no contraindications such as intracranial hypertension (ICH), unstable spinal fractures or sternal dehiscence with instability. So PPV was promptly conducted as the first-line therapy. Prior to PPV implementation given the early postoperative phase, we administered: Sedation: Midazolam to achieve RASS −3 to −5; Analgesia: Remifentanil infusion; Gut management: Enteral nutrition suspension >2 h, continuous gastric decompression during PPV. Concurrent safety measures: ①. Incision stabilization with thoracic binder; ②. Maintenance of drainage tube patency; ③. Endotracheal tube position verification; ④. Tracheal secretion suctioning. After 6 h of prone positioning ventilation, the P/F improved to 176 mmHg, further increasing to 225 mmHg at 10 h. INO was not administered concurrently at that time due to the significant oxygenation improvement achieved with PPV. However, upon returning to the supine positioning within 4 h, oxygenation declined again (P/F: 81.8 mmHg). Given concerns about potential wound complications from repeated PPV sessions within a short timeframe, iNO therapy was initiated at 10 ppm. Similar to Case 1, the imaging manifestations showed no significant changes on repeat chest radiography 2 days post iNo treatment (Figure 2B) in comparision with Figure 2A, but oxygenation progressively improved, reaching 140 mmHg at 6 h and 170 mmHg at 12 h after iNO administration. Subsequently, considering the potentially superior efficacy of PPV observed earlier, PPV was reintroduced, which resulted in a P/F increase from 104 mmHg to 161 mmHg after 10.5 h. As oxygenation significantly improved during PPV, iNO therapy was temporarily suspended during PPV sessions. Following prone session completion, iNO was resumed for 8 h, achieving a P/F of 293 mmHg (shown in Figure 2C).

Total prone positioning duration was 35 h (administered in 4 sessions), and iNO therapy spanned 41.5 h. The patient was successfully extubated on postoperative day 7 after achieveing the weaning criteria. The patient had stayed in ICU for10 days with total hospitalization of 16 days, then discharged from the hospital and returned home.

## Discussion

Hypoxemia after cardiopulmonary bypass for Type A Aortic Dissection is a common complication, especially in Sun's Procedure in Patients with Acute Type A Aortic Dissection, where the proportion of hypoxemia is higher (10). Most cases are related to ischemia-reconcern injury (10). The peak of its complication hypoxemia is often 24–48 h after the operation. Therefore, glucocorticoids are now routinely used during and after the operation in clinical practice to prevent and improve ischemic reconcern injury (10). In addition, an important factor is related to the decreased myocardial contractility after surgery, acute kidney injury, and fluid balance overload. Therefore, CRRT or diuretic is often used after surgery for strict fluid negative balance management (11). The third factor is the aggravation of preoperative pulmonary inflammation or the new pulmonary infection that occurs 2 to 3 days after the operation. Due to the ischemia-reperfusion injury of the lungs, the damage of the internal screen barrier, and mechanical ventilation, it is very easy for the pulmonary infection to worsen (12, 13). Therefore, the prophylactic use of broad-spectrum antibiotics after the operation is also an important part of postoperative management. The fourth factor is obesity-induced decreased lung compliance (10, 14). Optimal ventilator management constitutes one of the cardinal therapeutic interventions; Implemented a lung-protective ventilation strategy; Determined optimal PEEP through titration; Performed recruitment maneuvers (10, 15).

However, the two patients presented in this report demonstrated suboptimal responses to these standard interventions mentioned above. The studies show that if these approaches prove insufficient, PPV becomes one of the effective treatment options for patients with hypoxemia (16, 17). PPV improves oxygenation by optimizing ventilation distribution, reducing intrapulmonary shunting, and facilitating secretion drainage. Studies have demonstrated that PPV not only improves the oxygenation index in severe ARDS patients but also reduces mortality (5). However, applying PPV in ARDS patients following ATAAD surgery carries potential risks, including impaired wound healing, early hemodynamic instability, bleeding, catheter dislodgement, and even cardiac arrest (6). Therefore, PPV should be used cautiously in post-ATAAD ARDS patients. Prior to PPV implementation given the early postoperative phase, we first assessed the patient for contraindications to PPV, including: ICH or unstable spinal fractures, active bleeding, hemodynamic instability or sternal dehiscence with instability. Once contraindications were ruled out, then we administered: Sedation: Midazolam to achieve RASS −3 to −5; Analgesia: Remifentanil infusion; Administer neuromuscular blocking agents (NMBAs) intravenously as needed; Gut management: Enteral nutrition suspension >2 h, continuous gastric decompression during PPV. Concurrent safety measures: ①. Incision stabilization with thoracic binder; ②. Maintenance of drainage tube patency; ③. Endotracheal tube position verification; ④. Tracheal secretion suctioning. Next, PPV was initiated following institutional guidelines, with comprehensive post-positioning care to monitor clinical response and mitigate complications. In addition, NO exerts anti-inflammatory effects, mitigates ischemia/reperfusion (18), and alleviates pulmonary inflammation and injury (19, 20). It selectively dilates pulmonary vessels, improving ventilation-perfusion matching and oxygenation, though it does not reduce mortality. Therefore, We hypothesize that combined PPV and iNO therapy may have a synergistic effect in refractory hypoxemia following ATAAD surgery. This approach may not only improve oxygenation and reduce mortality risk but also shorten the duration and frequency of PPV, thereby decreasing the incidence of adverse complications.

In Case 1, a severely obese patient developed severe hypoxemia following aortic dissection surgery. In addition to conventional therapies, PPV combined with iNO therapy was implemented. PPV enhanced secretion drainage via gravitational clearance. Then fiberoptic bronchoscopy removed secretions and relieved airway obstruction. In additon, iNO improved ventilation/perfusion (V/Q) matching. A serious of these interventions significantly improved oxygenation, obviating the need for venovenous extracorporeal membrane oxygenation (V-V ECMO) and tracheotomy, with no adverse complications observed. In Case 2, another patient with postoperative hypoxemia following aortic dissection surgery exhibited limited improvement with PPV alone. During PPV, the patient demonstrated significant improvement in OI, therefore iNO therapy was not concurrently initiated. While during non-prone periods, oxygenation deteriorated again, making weaning difficult. Intermittent iNO therapy significantly improved oxygenation, facilitating successful weaning, transfer out of the ICU, and eventual discharge in good health.

Currently, the application of combined PPV and iNO therapy in ARDS patients following aortic dissection surgery has not been reported. Current guidelines recommend PPV for ARDS patients, they do not endorse iNO therapy due to its potential nephrotoxic effects (5). However, increasing recent evidence has demonstrated that iNO can effectively improve hypoxemia with minimal side effects (21, 22). During the COVID-19 pandemic, iNO was widely used and proven to improve oxygenation and shorten the length of ventilatory support in patients with ARDS caused by COVID-19.The mechanism of action was thought to be pulmonary vasodilation and consequent improved oxygenation in the blood of the lungs, thereby killing the virus (23, 24). In addition, iNO has shown significant therapeutic effects in post-aortic dissection ARDS without apparent adverse complications, which may be attributed to low-dose administration (5–20 ppm), as detailed in Table 1 (7–9, 25). Based on literature review and the two case studies presented in this article, the combination of PPV and iNO therapy may provide the following clinical benefits for patients with refractory hypoxemia after aortic dissection surgery: (1) improvement of intrapulmonary shunting to enhance oxygenation; (2) reduction in duration of mechanical ventilation; and (3) decreased incidence of adverse complications. However, the frequency and duration of prone positioning therapy, the dosage and duration of iNO therapy, as well as the mechanisms underlying the synergistic effects of combined prone positioning and iNO therapy in ATAAD patients still require further experimental exploration.

**Table 1:** Summary of the characteristics of the included studies.

Study (year)	Study type	Inclusion criteria	Sample (iNO group)	Sample (control group)	INO dosage (ppm)	Conclusion
Min Yu, et al., 2020	Article	Postoperative hypoxemia (PaO2/FiO2 ratio ≤200 mmHg) in AADA patients	40	94	5–10	Low-dose iNO improved oxygenation in patients with hypoxemia after AADA surgery and shortened the durations of mechanical ventilation and ICU stay
Luozhe et al., 2019	Article	Postoperative hypoxemia (PaO2/FiO2 ratio ≤100 mmHg) in AADA patients	26	27	5	INO therapy lead to sustained improvement in oxygenation and reduce the duration of invasive mechanical ventilation
CHO et al., 2022	Case	Severe Respiratory Failure after Acute Type A Aortic Dissection Surgery	1	N/A	20	The combination therapy of iNO and V-V ECMO for severe respiratory failure after surgery for ATAAD is effective
Luozhe et al., 2021	Article	Postoperative hypoxemia (PaO2/FiO2 ratio ≤150 mmHg) in AAAD patients	20	N/A	5	INO, rather than increasing PEEP, significantly decreased the intrapulmonary shunt to improve severe hypoxemic conditions.
Our current study	Case	Patients with Refractory Hypoxemia Following Acute Type A Aortic Dissection Surgery	2	N/A	5–10	The combined strategy of iNO and PPV shows potential promise for treating cases of refractory hypoxemia and merits further clinical investigation

AADA, acute type A aortic dissection; iNO, inhaled nitric oxide; PPV, prone posioning ventilaton; N/A, Not avaible.

## Conclusion

Our cases highlight the therapeutic potential of combined PPV and iNO for managing refractory hypoxemia following acute type A aortic dissection surgery, particularly in patients unresponsive to conventional therapies. The optimal dosage of iNO, duration of PPV, and strategies for their combined application require further clinical investigation.

## Data Availability

The datasets presented in this study can be found in online repositories. The names of the repository/repositories and accession number(s) can be found in the article/Supplementary Material.
